# Characterization of tumor-associated T-lymphocyte subsets and immune checkpoint molecules in head and neck squamous cell carcinoma

**DOI:** 10.18632/oncotarget.17901

**Published:** 2017-05-16

**Authors:** Axel Lechner, Hans Schlößer, Sacha I. Rothschild, Martin Thelen, Sabrina Reuter, Peter Zentis, Alexander Shimabukuro-Vornhagen, Sebastian Theurich, Kerstin Wennhold, Maria Garcia-Marquez, Lars Tharun, Alexander Quaas, Astrid Schauss, Jörg Isensee, Tim Hucho, Christian Huebbers, Michael von Bergwelt-Baildon, Dirk Beutner

**Affiliations:** ^1^ Cologne Interventional Immunology, Department I of Internal Medicine, University Hospital of Cologne, Cologne, Germany; ^2^ Department of Otorhinolaryngology, Head and Neck Surgery, University of Cologne, Cologne, Germany; ^3^ Department of General, Visceral and Cancer Surgery, University of Cologne, Cologne, Germany; ^4^ University Hospital Basel, Department of Internal Medicine, Medical Oncology, Basel, Switzerland; ^5^ Cologne Excellence Cluster on Cellular Stress Responses in Aging-Associated Diseases (CECAD), Cologne, Germany; ^6^ Department I of Internal Medicine, Center for Integrated Oncology (CIO), University Hospital of Cologne, Cologne, Germany; ^7^ Max-Planck-Institute for Metabolism Research, Cologne, Germany; ^8^ Institute of Pathology, University of Cologne, Cologne, Germany; ^9^ Department of Anesthesiology and Intensive Care Medicine, Experimental Anesthesiology and Pain Research, University Hospital of Cologne, University of Cologne, Germany; ^10^ Jean-Uhrmacher Institute for Clinical ENT Research, University of Cologne, Cologne, Germany

**Keywords:** squamous cell carcinoma, head and neck, microenvironment, T cells, immune checkpoint

## Abstract

The composition of tumor-infiltrating lymphocytes (TIL) reflects biology and immunogenicity of cancer. Here, we characterize T-cell subsets and expression of immune checkpoint molecules in head and neck squamous cell carcinoma (HNSCC). We analyzed TIL subsets in primary tumors (*n* = 34), blood (peripheral blood mononuclear cells (PBMC); *n* = 34) and non-cancerous mucosa (*n* = 7) of 34 treatment-naïve HNSCC patients and PBMC of 15 healthy controls. Flow cytometry analyses revealed a highly variable T-cell infiltration mainly of an effector memory phenotype (CD45RA^−^/CCR7^−^). Naïve T cells (CD45RA^+^/CCR7^+^) were decreased in the microenvironment compared to PBMC of patients, while regulatory T cells (CD4^+^/CD25^+^/CD127^low^ and CD4^+^/CD39^+^) were elevated. Furthermore, we performed digital image analyses of entire cross sections of HNSCC to define the ‘Immunoscore’ (CD3^+^ and CD8^+^ cell infiltration in tumor core and invasive margin) and quantified MHC class I expression on tumor cells by immunohistochemistry. Immune checkpoint molecules cytotoxic T-lymphocyte-associated antigen 4 (CTLA-4), programmed cell death 1 (PD-1) and programmed cell death 1 ligand 1 (PD-L1) were increased in TILs compared to peripheral T cells in flow-cytometric analysis. Human papillomavirus (HPV) positive tumors showed higher numbers of TILs, but a similar composition of T-cell subsets and checkpoint molecule expression compared to HPV negative tumors. Taken together, the tumor microenvironment of HNSCC is characterized by a strong infiltration of regulatory T cells and high checkpoint molecule expression on T-cell subsets. In view of increasingly used immunotherapies, a detailed knowledge of TILs and checkpoint molecule expression on TILs is of high translational relevance.

## INTRODUCTION

Head and neck squamous cell carcinoma (HNSCC) is in most cases related to smoking and alcohol consumption [1]. However, the incidence of HNSCC associated with human papillomavirus (HPV) as an independent risk factor is increasing [2]. HPV positive HNSCC are associated with distinct clinical characteristics showing better response to chemoradiotherapy and improved prognosis compared to HPV negative tumors [3]. One explanation for the differing behavior of these two entities would be the difference in their tumor microenvironment with a distinct composition of tumor-infiltrating lymphocytes (TILs) that may facilitate an improved adaptive host immune response against viral antigens [4].

In recent years, it has been well established that the immune system plays a key role in the control of tumor growth and progression [5, 6]. Tumor-infiltrating immune cells, including T and B lymphocytes, macrophages or neutrophils can either inhibit or enhance tumor growth. Our group recently investigated TILs in different solid tumors [7, 8]. Regulatory T cells (Treg) are responsible for the control of autoreactive lymphocytes, but also downregulate the immune response to tumor-associated antigens. Presence of Treg has been reported among TILs and in the peripheral circulation in a variety of solid tumors [9–12]. In patients with HNSCC, the antitumor immune response is impaired and progression is associated with severe immune dysfunction [13]. However, infiltration by certain immune-effector cells is associated with favorable outcome [14]. Nevertheless, inconsistent results have been reported especially for Treg subsets [15, 16]. Galon *et al*. established a strong correlation between type, density and location of TILs and the prognosis of cancer [17, 18]. They proposed a standardized algorithm to determine antitumor immune responses using quantitative pathology. This ‘Immunoscore’ is based on the quantification of CD3^+^ and CD8^+^ TILs in the tumor core (CT) and the invasive margin (IM) and has proven to be a strong prognostic tool in colorectal cancer [19, 20]. To date, an automated ‘Immunoscore’ analysis on entire cross sections of HNSCC has not been applied. Recently, it was demonstrated that immune cell infiltration patterns in non-small cell lung cancer differ depending on the degree of major histocompatibility complex (MHC) class I expression on tumor cells [21]. Furthermore, high MHC class I expression on tumor cells is associated with improved disease-free survival (DFS) in patients with HPV negative tonsillar squamous cell carcinoma compared to low MHC class I expression. However, this association is inverse in HPV positive tonsillar carcinoma [22].

Immune checkpoint molecules including cytotoxic T-lymphocyte-associated antigen 4 (CTLA-4), programmed cell death 1 (PD-1) and programmed cell death 1 ligand 1 (PD-L1) contribute to an immunosuppressive microenvironment in a variety of solid cancers. They can be expressed by several cells and are key molecules also for Treg [23, 24]. Specific antibodies targeting these molecules have recently changed the therapeutic landscape not only in head and neck cancer treatment [25].

A detailed characterization of the composition of the tumor microenvironment and of checkpoint molecule expression is crucial to understand mechanisms of immunotherapies.

## RESULTS

### CD45^+^ lymphocyte count is highly variable in HNSCC

**Figure 1:**
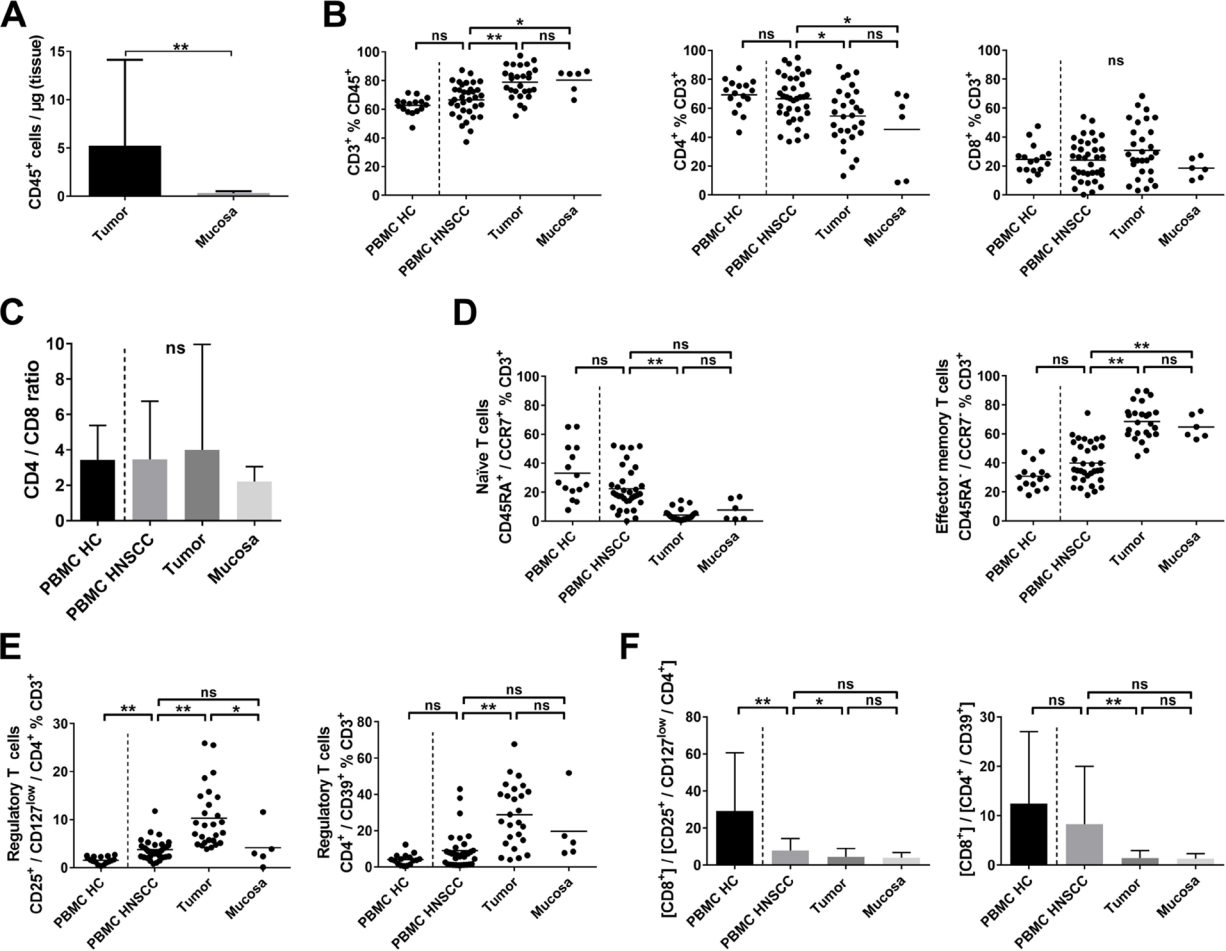
T-cell subsets in PBMC, tumor samples and non-cancerous mucosa of HNSCC patients and PBMC of healthy controls Single cell suspensions of tumor tissue (*n* = 34), non-cancerous mucosa (*n* = 7), PBMCs of healthy controls (PBMC HC, *n* = 15) and PBMCs of patients with HNSCC (PBMC HNSCC, *n* = 34) were analyzed by flow cytometry for their expression of T-cell related antigens. (**A**) Scatter plots showing the number of CD45^+^ cells per μg of tumor or mucosal tissue. (**B**) Scatter plots comparing the percentages of CD3^+^ T cells within the CD45^+^ fraction (left), CD4^+^ (middle plot) and CD8^+^ (right) cells within the T cells fraction. (**C**) Depicted in bar graphs is the ratio of CD4^+^ and CD8^+^ T cells (CD3^+^ fraction) in different compartments. (**D**) Comparison of the rate of naïve (CD45RA^+^/CCR7^+^; left) and effector memory T cells (CD45RA^−^/CCR7^−^; right) in the T-cell fraction, shown as scatter plots. (**E**) Percentages of regulatory T-cell phenotypes CD4^+^/CD25^+^/CD127^low^ and CD4^+^/CD39^+^ within PBMC of healthy donors, HNSCC patients, HNSCC tumor tissue and normal mucosa are compared in scatter plots. (**F**) Bar graphs comparing the ratio of CD8^+^ cells and regulatory T-cell phenotypes CD4^+^/CD25^+^/CD127^low^ and CD4^+^/CD39^+^ within the CD3^+^ fraction. For statistical analysis, Mann-Whitney test was used in (A), one-way ANOVA in (B) and right plot of (D) and Kruskal-Wallis test in (C), left plot of (D), (E) and (F). Data is depicted as mean ± standard deviation. *P < 0.05; **P < 0.005; ns, not significant.

We assessed the composition of TIL populations in tumor tissue of 34 previously untreated HNSCC patients (26 HPV negative tumors, 7 HPV-16-associated HNSCC, 1 HPV-35-associated HNSCC) by flow cytometry. Sample acquisition was conducted prior to initiation of anticancer treatment. Clinical characteristics of included patients are summarized in Table 1. The absolute number of CD45^+^ lymphocytes per microgram tissue was highly heterogeneous in HNSCC, ranging from 0.1 cells/μg to 44.2 cells/μg with an average of 5.2 ± 8.9 cells/μg, which was significantly higher than the number of CD45^+^ cells in randomly biopsied non-cancerous mucosa samples (*n* = 7, 0.3 ± 0.2/μg; *p* = 0.004; Figure 1A).

**Table 1:** Patient and healthy donor characteristics

Characteristics	*N*	%
HNSCC patients (*n* = 34)		
Median age (range)	68 (49–85)	
Sex		
Male	27	79%
Female	7	21%
Localisation		
Oral cavity	5	14.7%
Oropharynx	16	47.1%
Hypopharynx	5	14.7%
Larynx	7	20.6%
Other (nasal cavity)	1	2.9%
UICC stage		
I	2	5.9%
II	9	26.5%
III	6	17.6%
IVA	16	47.1%
IVB	0	0.0%
IVC	1	2.9%
Histological grading		
G1	0	0
G2	28	82%
G3	6	18%
HPV status		
positive	8	24%
negative	26	76%
Healthy donors (*n* = 15)		
Median age (range)	61 (43-79)	
Sex		
Male	12	80%
Female	3	20%

HNSCC = head and neck squamous cell carcinoma; HPV = human papillomavirus; RT = radiotherapy; UICC = Union for International Cancer Control.

### Tumor-infiltrating T cells are mainly of a CD45RA^−^/CCR7^−^ effector memory phenotype, while Treg are significantly increased

T cells accounted for 78.8 ± 10.9% of CD45^+^ lymphocytes in tumor samples compared to 80.3 ± 8.1% in non-cancerous mucosa, 62.7 ± 5.9% in peripheral blood mononuclear cells (PBMC) of aged-matched healthy controls (PBMC HC, *n* = 15) and 66.6 ± 11.7% in peripheral blood samples of HNSCC patients (PBMC HNSCC; Figure 1B, left plot). No significant difference was detected in the percentage of CD8^+^ cytotoxic T cells in tumor samples (30.9 ± 18.7% of T cells) compared to non-cancerous mucosa (18.5 ± 6.9%), PBMC HC (24.6 ± 9.9%) or PBMC HNSCC (24.0 ± 14.0%; Figure 1B, right plot). However, the percentage of CD4^+^ T cells was significantly decreased in tumor samples (54.7 ± 19.6%) and mucosa (45.3 ± 28.6%) compared to PBMC HNSCC (66.6 ± 15.9%; *p <* 0.05; Figure 1B, middle plot). Comparable percentages of CD4^+^ T cells were observed in PBMC HNSCC and PBMC HC (66.6 ± 15.9% vs. 69.3 ± 11.1%). The CD4/CD8 ratio did not differ between all groups (Figure 1C).

Whereas naïve T cells (CD45RA^+^/CCR7^+^) constituted 33.2 ± 18.3% of T cells in the peripheral blood of healthy controls, their percentage in PBMC HNSCC was 22.4 ± 14.6%, in tumor samples 4.1 ± 3.8% and in non-cancerous mucosa 7.7 ± 7.2% (Figure 1D, left plot). The percentage in tumor samples was significantly decreased compared to PBMC HNSCC (*p <* 0.0001). Mucosa-associated T cells were predominantly antigen-experienced CD45RA^−^/CCR7^−^ effector memory T cells with 68.6 ± 12.3% of tumor-infiltrating T cells and 64.7 ± 8.2% of T cells in non-cancerous mucosa compared to significantly lower 39.9 ± 13.9% in PBMC HNSCC (*p <* 0.0001; Figure 1D, right plot). Percentages of effector memory T cells in PBMC of healthy donors and HNSCC patients were comparable (30.8 ± 9.3% vs. 39.9 ± 13.9%).

To assess regulatory T cells, we analyzed the CD4^+^/CD25^+^/CD127^low^ and the CD4^+^/CD39^+^ T-cell subset, which have been reported to contain a high percentage of FoxP3^+^ Treg [26–28]. CD4^+^/CD25^+^/CD127^low^ Treg made up a significantly larger proportion of T cells in tumor samples (10.3 ± 6.3%) compared to PBMC HNSCC (3.7 ± 2.0%; *p <* 0.0001) and non-cancerous mucosa (4.2 ± 4.4%, *p <* 0.05; Figure 1E, left plot). The proportion of this regulatory T-cell phenotype was significantly increased in PBMC derived from HNSCC patients compared to healthy donors (3.7 ± 2.0% vs. 1.6 ± 0.7%; *p <* 0.005). Significant differences in the CD4^+^/CD39^+^ T-cell population were detected in tumor tissue (28.8 ± 17.1% of T cells) compared to PBMC HNSCC (8.9 ± 10.2%, *p <* 0.0001), but not compared to non-cancerous mucosa (19.7 ± 18.4%). The difference between PBMC HNSCC and PBMC HC was statistically not significant (8.9 ± 10.2% vs. 4.2 ± 3.1%, *p* = 0.38; Figure 1E, right plot). The immunosuppressive microenvironment is also reflected by a low CD8/Treg ratio. [CD8^+^] / [CD4^+^/CD25^+^/CD127^low^] ratio was similarly decreased in tumor (4.4 ± 4.5) and mucosa (4.0 ± 2.7), being statistically significant between tumor samples and PBMC HNSCC (4.4 ± 4.5 vs. 7.8 ± 6.4; *p <* 0.05). Comparison between PBMC HNSCC and PBMC HC revealed a significant decrease of the [CD8^+^] / [CD4^+^/CD25^+^/CD127^low^] ratio in PBMC of HNSCC patients (7.8 ± 6.4 vs. 29.1 ± 31.5; *p <* 0.005; Figure 1F, left plot). However, [CD8^+^] / [CD4^+^/CD39^+^] ratio only differed significantly between tumor tissue and PBMC HNSCC (1.4 ± 1.5 vs. 8.3 ± 11.8; *p <* 0.005; Figure 1F, right plot).

### Digital analysis of CD3^+^ and CD8^+^ cells in HNSCC (immunohistochemistry) shows high concordance with flow-cytometric results and increased MHC I expression is associated with high T-cell infiltration

**Figure 2:**
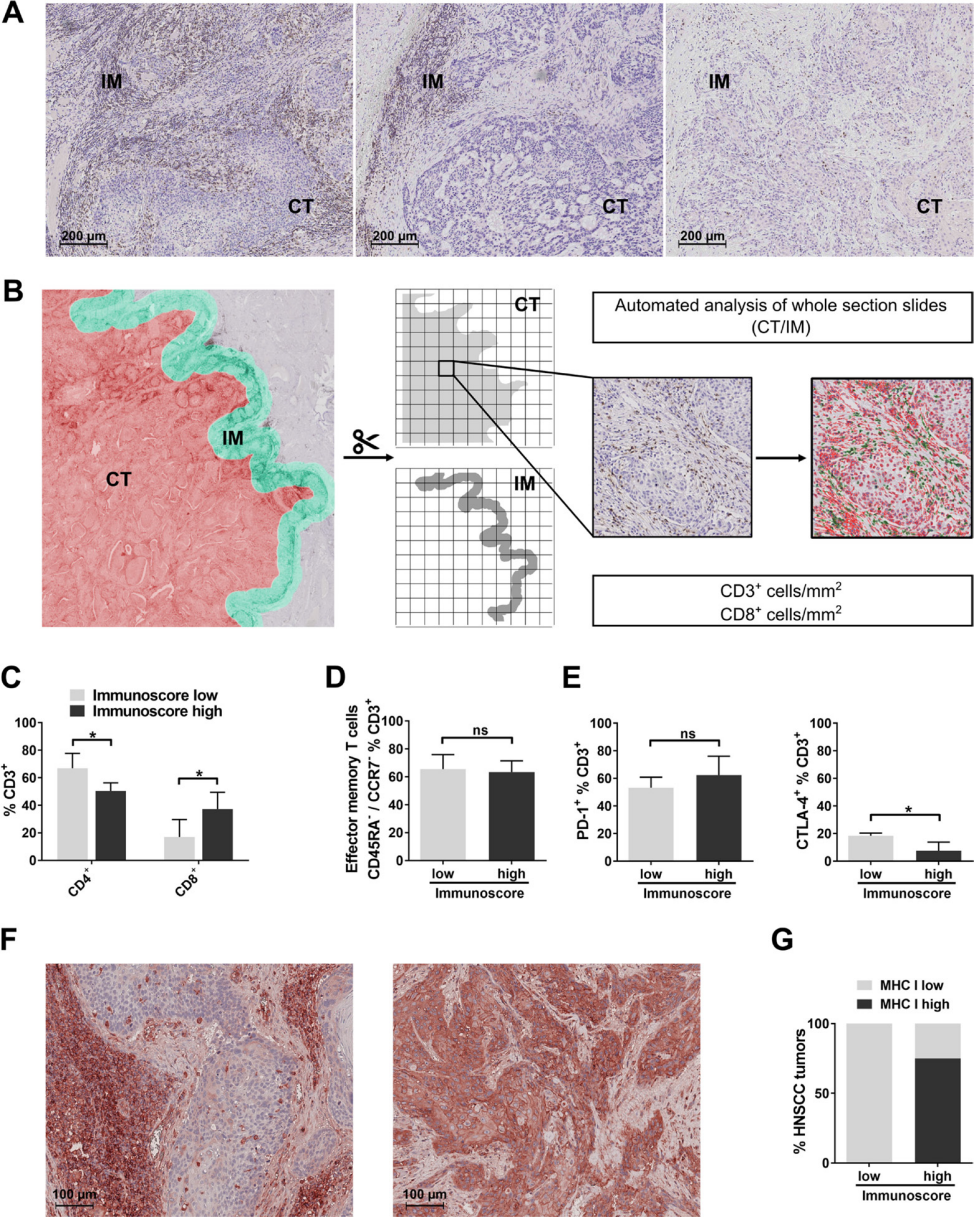
Digital analysis of CD3^+^ and CD8^+^ cells in cross sections of entire HNSCC tumors and comparison with results obtained by flow cytometry (**A**) Exemplary immunohistochemical staining of CD3 showing three different T-cell infiltration patterns in tumor core (CT) and invasive margin (IM) of HNSCC: (1) high CD3^+^ and CD8^+^ cell infiltration of IM and CT, (2) high TIL density in the IM and (3) low CD3^+^ and CD8^+^ infiltration in CT and IM. (**B**) Analysis process of immunohistochemical stainings of entire HNSCC tumor cross sections: cross sections of HNSCC stained for CD3 and CD8 are subjected to automated analysis of IM and CT. IM (area ranging from 50 μm within the tumor to 300 μm outside the tumor border; green area) and CT (whole tumor section excluding first 50 μm adjacent to the tumor border; red area) are schematically shown on the left side. Both areas are subsequently separated and cropped into tiles suitable for automated analysis of DAB-stained cells. Shown on the right side is an exemplary tile derived from the CT stained for CD3 before and after analysis (positive cells are displayed in green, nuclei in red). As a result, CD3^+^ and CD8^+^ cell count per mm^2^ is calculated for the whole area of IM and CT, respectively. (**C**) Bar graphs showing flow-cytometrically determined percentages of CD4^+^ and CD8^+^ T cells in ‘Immunoscore low’ and ‘Immunoscore high’ HNSCC. (**D**) Comparison of percentages of effector memory T cells in ‘Immunoscore low’ and ‘Immunoscore high’ tumors. (**E**) PD-1 (left) and CTLA-4 (right) expression on T cells compared in ‘Immunoscore low’ and ‘Immunoscore high’ HNSCC. (**F**) Examples of low (left) and high (right) MHC class I expression of HNSCC tumor cells in immunohistochemical stainings. (**G**) Stacked columns showing percentages of high and low MHC class I expression in HPV negative HNSCC classified as ‘Immunoscore low’ (*n* = 6) and ‘Immunoscore high’ (*n* = 4). The probability for high MHC I expression in tumor cells is significantly higher in the ‘Immunoscore high’ than the ‘Immunoscore low’ group (*p <* 0.05; Fisher's exac*t* test). In (C) - (E), Student's *t* test was performed (‘Immunoscore high’, *n* = 5; ‘Immunoscore low’, *n* = 6). Data is depicted as mean ± standard deviation. *P < 0.05; ns, not significant.

Our customized digital image analysis protocol based on CellProfiler enabled us to perform quantification of CD3^+^ and CD8^+^ cells in entire cross sections of HNSCC tumors. Three different patterns of CD3^+^ and CD8^+^ cell infiltration could be discriminated: (1) high CD3^+^ and CD8^+^ cell density in tumor core (CT) and invasive margin (IM), (2) high CD3^+^ and CD8^+^ cell density in IM and low density in CT and (3) low CD3^+^ and CD8^+^ cell density in CT and IM as shown by CD3 immunohistochemistry in three different exemplary HNSCC (Figure 2A). A schematic overview of the digital analysis process is depicted in Figure 2B. Results obtained by automated analysis were highly concordant with optical cell counting of selected slides. On average, an area of 5.4 mm^2^ (IM CD3), 25.6 mm^2^ (CT CD3), 5.0 mm^2^ (IM CD8) and 26.1 mm^2^ (CT CD8) was analyzed per section. Tumors with high CD3^+^ and CD8^+^ cell infiltration classified as ‘Immunoscore high’ (*n* = 5) showed a mean CD3^+^ and CD8^+^ cell count of 551/mm^2^ ± 215/mm^2^ and 318/mm^2^ ± 127/mm^2^, respectively, within the CT. CD3^+^ and CD8^+^ cell densities were significantly increased in the IM (1117/mm^2^ ± 188/mm^2^ and 784/mm^2^ ± 66/mm^2^, respectively) compared to the CT (*p <* 0.005). In contrast, CD3^+^ and CD8^+^ cell densities in the CT in tumors classified as CD3^+^ low and CD8^+^ low (‘Immunoscore low’; *n* = 6) was 173/mm^2^ ± 160/mm^2^ and 50/mm^2^ ± 22/mm^2^ compared to 621/mm^2^ ± 327/mm^2^ and 247/mm^2^ ± 151/mm^2^ in the IM, again showing higher CD3^+^ and CD8^+^ cell infiltration in the IM than in the CT (*p <* 0.05; Supplementary Figure 1). No HPV positive HNSCC was classified as ‘Immunoscore low’.

We then compared tumor-infiltrating T-cell subsets determined by flow cytometry in ‘Immunoscore high’ and ‘Immunoscore low’ tumors. Compared to tumors which were classified as ‘Immunoscore high’ in immunohistochemical analyses, ‘Immunoscore low’ HNSCC tumors showed a significantly higher percentage of CD4^+^ T cells (67.0 ± 10.7% vs. 50.5 ± 5.9%; *p <* 0.05) and significantly lower percentages of CD8^+^ T cells (16.9 ± 12.8% vs. 37.3 ± 12.0%; *p <* 0.05; Figure 2C) in flow cytometry analyses. However, no differences were detected regarding the flow cytometrically determined percentage of effector memory T cells between ‘Immunoscore low’ and ‘Immunoscore high’ populations (65.6 ± 10.3% vs. 63.4 ± 8.1%; Figure 2D). PD-1^+^ T cells were not significantly increased in ‘Immunoscore high’ compared to ‘Immunoscore low’ tumors (62.4 ± 13.7% vs. 53.3 ± 7.6%; *p* = 0.31), while CTLA-4^+^ T cells were significantly decreased (7.5 ± 6.3% and 18.4 ± 2.0%, respectively; *p <* 0.05; Figure 2E). Regulatory T-cell populations (CD4^+^/CD39^+^; CD4^+^/CD25^+^/CD127^low^) showed similar percentages in both groups (data not shown).

It has been shown in non-small cell lung cancer that high intratumoral T-cell infiltration occurs mainly in tumors with high expression levels of MHC class I on tumor cells [21]. We therefore examined MHC class I expression in HNSCC previously classified as ‘Immunoscore high’ and ‘Immunoscore low’. As it is known that in HPV 16 positive cancer cells viral oncoprotein E7 leads to a decreased MHC class I expression via transcriptional regulation [29], only HPV negative HNSCC were included. Different patterns of MHC I expression on tumor cells could be observed. An exemplary picture of a HNSCC tumor with low expression of MHC I on tumor cells, but high expression on surrounding and infiltrating immune cells is shown on the left, an example of high MHC I expression on tumor cells is shown on the right side of Figure 2F. ‘Immunoscore low’ HNSCC demonstrated low levels of MHC class I expression in 6/6 cases, whereas only 1/4 cases of ‘Immunoscore high’ tumors showed low and 3/4 cases high MHC I expression. The probability for high MHC I expression in tumor cells was significantly increased in the ‘Immunoscore high’ compared to the ‘Immunoscore low’ group (*p <* 0.05; Figure 2G). MHC I expression in HPV positive HNSCC was low in 4/5 cases (data not shown).

### PD-1, PD-L1 and CTLA-4 are highly expressed on T cells in HNSCC tumor tissue

**Figure 3:**
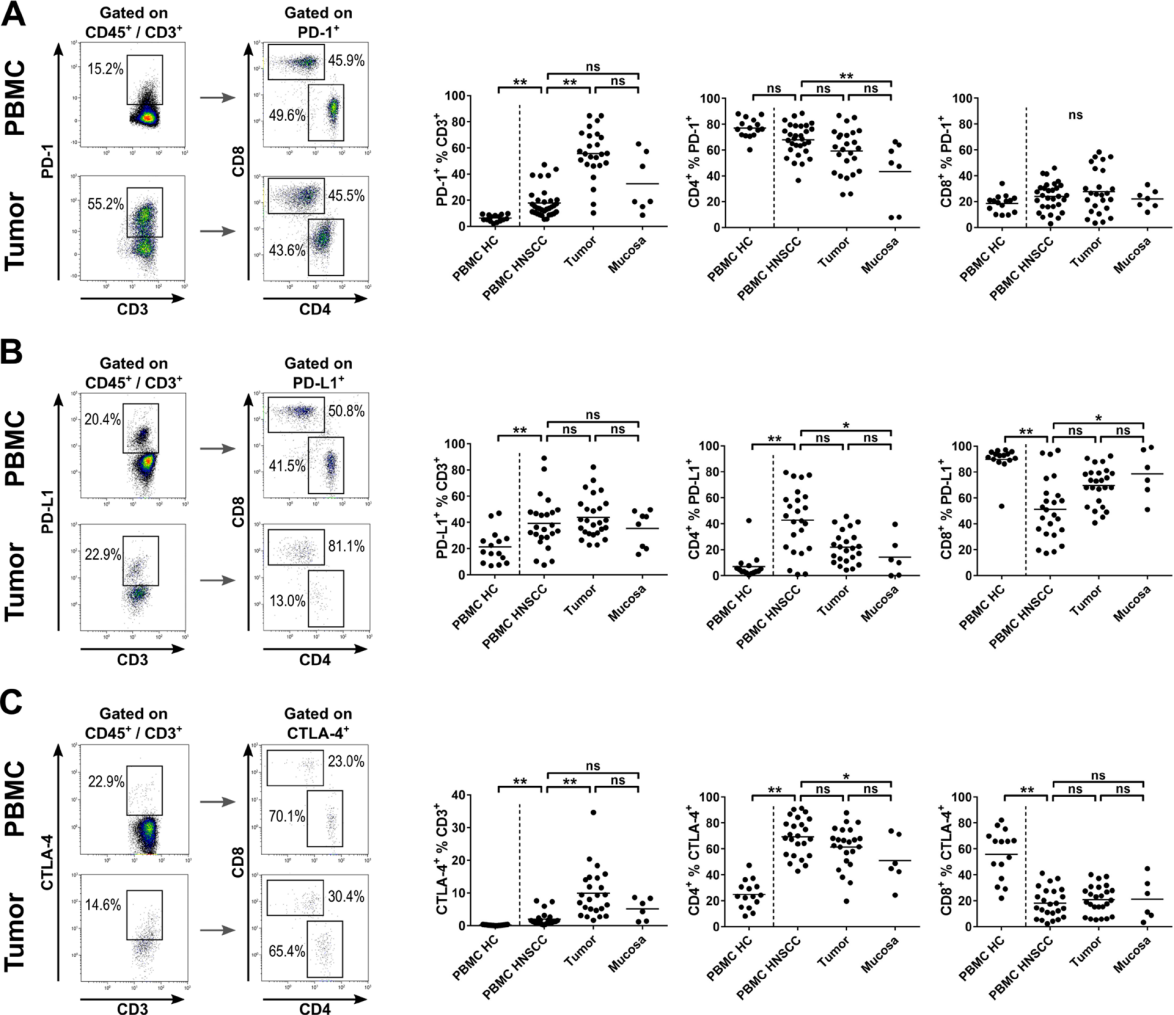
Expression of immune checkpoint molecules on T cells in HNSCC patients T-cell subsets in PBMCs of healthy controls (PBMC HC, *n* = 15), PBMCs of HNSCC patients (PBMC HNSCC, *n* = 32), single cell suspensions of tumor tissue (*n* = 31) and non-cancerous mucosa (*n* = 7) were analyzed by flow cytometry for immune checkpoint molecule expression. (**A**) Exemplary flow cytometry analysis of PD-1 expression in PBMC and tumor tissue of HNSCC. PD-1 expressing T cells were subcategorized according to their membranous CD4 and CD8 expression, as shown in dot plot graphs on the left side. Scatter plots comparing the percentage of PD-1 expressing cells within the T-cell fraction in PBMC HC, PBMC HNSCC, tumor tissue and mucosa (middle). The two scatter plot graphs on the right side show the percentage of CD4^+^ and CD8^+^ T cells within the PD-1^+^ T-cell fraction. (**B**) Similar to (A), exemplary dot plots of PD-L1 expressing T-cell subsets are depicted on the left side. Percentages of PD-L1 expressing T cells and CD4^+^ and CD8^+^ PD-L1^+^ T-cell subsets are shown in scatter plots on the right side (comparing PBMC HC, PBMC HNSCC, tumor tissue and mucosa in each graph). (**C**) Similar to (A), gating of CTLA-4^+^ T cells and subsets (CD4^+^ and CD8^+^) is shown on the left side. The percentage of CTLA-4 expressing T cells and CD4^+^ and CD8^+^ subsets within this fraction are depicted in scatter plots on the right side. For statistical analysis, one-way ANOVA was used in middle and right plot of (A) and (C) and left plot of (B). Kruskal-Wallis test was used for all remaining analyses. The mean of every dataset is depicted. *P < 0.05; **P < 0.005; ns, not significant.

In view of the increasing use of immune checkpoint inhibitors, we examined expression of PD-1, PD-L1 and CTLA-4 on T cells. HNSCC tumor tissue samples contained a significantly increased fraction of PD-1 expressing T cells (55.7 ± 19.0%) compared to PBMC HNSCC (18.0 ± 11.2%; *p <* 0.0001), but not compared to non-cancerous mucosa (32.6 ± 22.2%). An increased proportion of PD-1^+^ T cells was observed in PBMC HNSCC compared to PBMC HC (18.0 ± 11.2% vs. 6.4 ± 2.5%; *p <* 0.005). PD-1^+^ T cells were mainly CD4^+^, with 77.0 ± 7.5% in PBMC HC not significantly more frequent than in PBMC HNSCC (68.0 ± 12.2%). Percentages in non-cancerous mucosa were reduced compared to PBMC HNSCC (43.3 ± 25.1% vs. 68.0 ± 12.2%; *p <* 0.005). The difference between PBMC HNSCC and tumor tissue (59.3 ± 17.3%) was statistically not significant (*p* = 0.13). Percentages of CD8^+^ cytotoxic T cells in the PD-1^+^ fraction were comparably low in PBMC HNSCC (23.9 ± 10.9%), tumor tissue (27.8 ± 17.3%), non-cancerous mucosa (22.0 ± 7.2%) and PBMC HC (18.7 ± 6.7%; Figure 3A).

PD-L1 expression was significantly increased in the T-cell fraction of PBMC HNSCC (39.2 ± 19.9%) compared to PBMC HC (21.3 ± 12.6%; *p <* 0.05). PBMC HNSCC, tumor tissue (44.0 ± 15.9%) and non-cancerous mucosa (35.5 ± 14.0%) showed comparable percentages of PD-L1 expression within the T-cell fraction. Whereas in PBMC HC, CD4^+^ T cells made up only a small fraction of PD-L1^+^ T cells (7.1 ± 10.3%), the percentage highly increased in PBMC HNSCC (42.8 ± 24.4%; *p <* 0.0001). Non-cancerous mucosa (14.2 ± 15.1%) and tumor tissue (21.9 ± 12.4%) showed similar percentages of CD4^+^ T cells in the PD-L1^+^ fraction. The vast majority of PD-L1^+^ T cells in PBMC HC were cytotoxic T cells (89.8 ± 10.5%), with significantly lower percentages in PBMC HNSCC (51.1 ± 23.7%; *p <* 0.0001). Again, similar percentages of PD-L1^+^ cells were CD8^+^ in tumor tissue (69.6 ± 14.7%) and non-cancerous mucosa (78.5 ± 18.6%; Figure 3B).

CTLA-4^+^ T cells were substantially less frequent than PD-1^+^ and PD-L1^+^ T cells. Almost no CTLA-4 expressing T cells were observed in PBMC HC (0.2 ± 0.1%), which was significantly lower than the percentage detected in PBMC HNSCC (2.0 ± 2.1%, *p <* 0.005). However, the percentage of CTLA-4^+^ T cells increased significantly in HNSCC tumor microenvironment (10.0 ± 7.5%; *p <* 0.0001). The percentage of CTLA-4^+^ T cells in tumor tissue and non-cancerous mucosa (5.1 ± 3.3%) did not differ significantly. CD4^+^ T cells in PBMC HC made up a significantly lower proportion of CTLA-4^+^ T cells (24.8 ± 10.7%) than in PBMC HNSCC (69.3 ± 14.6%; *p <* 0.0001), whereas percentages in PBMC HNSCC and tumor tissue (61.4 ± 16.3%) were comparably high. The proportion of CD4^+^ cells among CTLA-4^+^ T cells in non-cancerous mucosa was decreased compared to PBMC HNSCC (51.0 ± 18.8% vs. 69.3 ± 14.6%; *p <* 0.05), but not compared to tumor tissue. The majority of CTLA-4^+^ T cells was CD8^+^ in PBMC HC (55.9 ± 19.1%), with significantly lower percentages of CD8^+^ T cells in the CTLA-4^+^ T-cell subset in PBMC HNSCC (18.2 ± 11.4%; *p <* 0.0001). Percentages in PBMC HNSCC, tumor tissue (20.9 ± 10.9%) and non-cancerous mucosa (21.2 ± 16.1%) were comparable (Figure 3C).

### HPV positive tumors show a higher number of tumor-infiltrating T cells, but a similar distribution of T-cell subsets compared to HPV negative tumors

**Figure 4:**
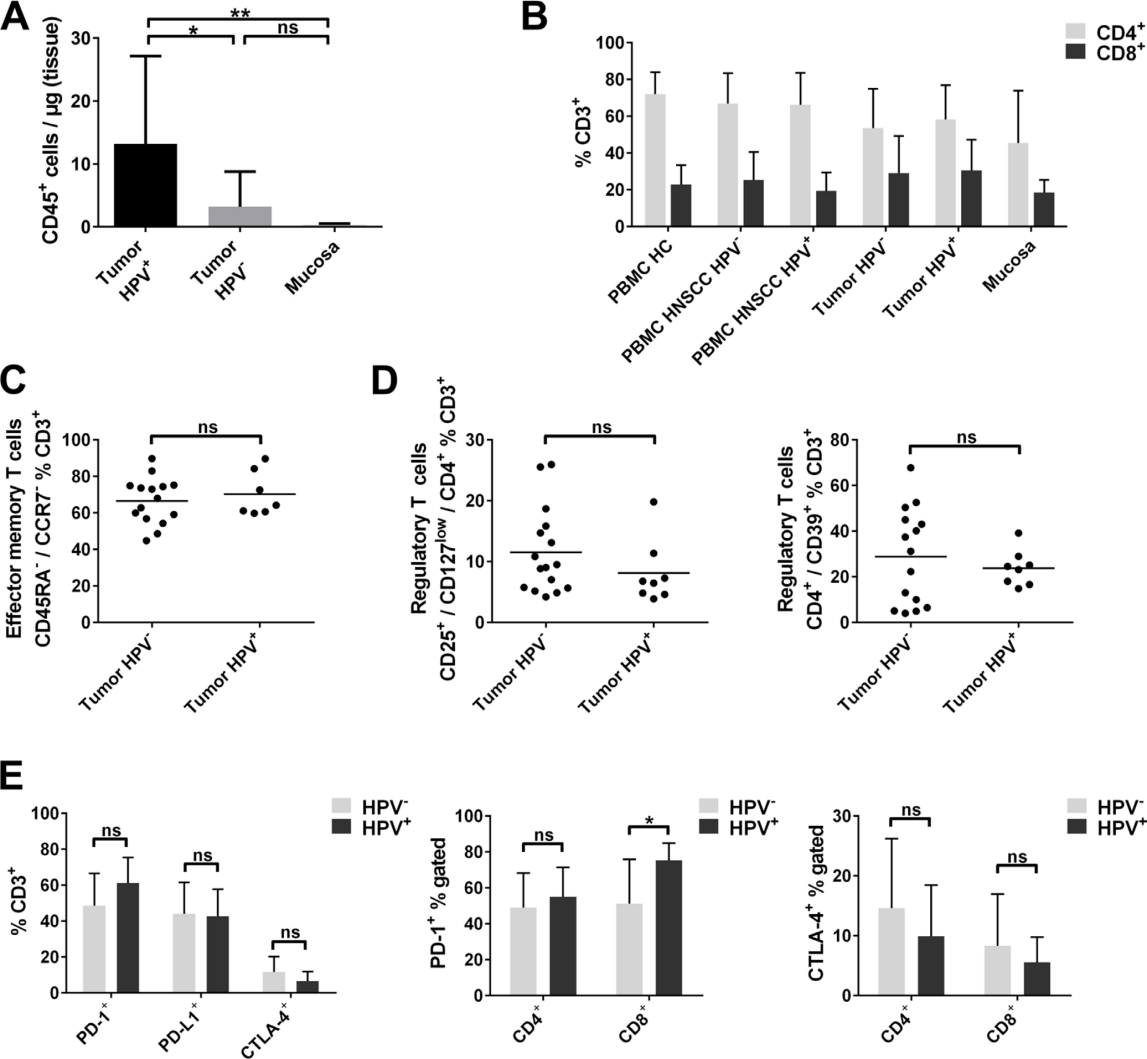
Lymphocyte infiltration and T-cell subsets in HPV positive and HPV negative HNSCC (**A**) Flow-cytometric analysis of CD45^+^ cells per μg tissue in HPV positive HNSCC tumors (*n* = 8), HPV negative HNSCC (*n* = 26) and non-cancerous mucosa of the same patients (*n* = 7). (**B**) Percentages of CD4^+^ and CD8^+^ cell subsets within the CD3^+^ T cells compared in PBMC of healthy donors, PBMC and tumor tissue of HPV negative and HPV positive HNSCC patients and non-cancerous mucosa. (**C**) Effector memory T-cell subsets (CD45RA^−^/CCR7^−^) are compared in tumor tissue of HPV positive and negative HNSCC. (**D**) Comparison of percentages of different regulatory T-cell phenotypes CD4^+^/CD25^+^/CD127^low^ and CD4^+^/CD39^+^ in HPV positive and negative HNSCC tumor tissue. (**E**) Overview of checkpoint molecule expressing T cells (PD-1, PD-L1 and CTLA-4) in the tumor microenvironment of HPV positive and negative HNSCC (left plot); percentages of PD-1^+^ and CTLA-4^+^ T cells in the CD4^+^ and CD8^+^ T-cell fraction of HPV positive and negative HNSCC tumor tissue (middle and right plot). Kruskal-Wallis test was used in (A) and *t* tests were performed in (C), (D) and (E). Data is depicted as mean ± standard deviation. *P < 0.05; **P < 0.0001; ns, not significant.

In HPV positive tumors, a higher number of CD45^+^ lymphocytes (13.2 ± 13.9 cells/μg) than in HPV negative tumors (3.2 ± 5.6 cells/μg; *p <* 0.05) and in non-cancerous mucosa (0.3 ± 0.2 cells/μg; *p <* 0.0001) was detected (Figure 4A). Comparable results were obtained when analyzing oropharyngeal squamous cell carcinoma only (data not shown). We did not find any significant differences in the rate of CD4^+^ or CD8^+^ T cells related to the HPV status (Figure 4B). The proportion of effector memory T cells was similarly high in the tumor microenvironment of HPV positive and HPV negative patients (66.5 ± 12.7% and 70.2 ± 12.2%; Figure 4C). There was a statistically not significant trend towards higher numbers of regulatory T cells in the microenvironment of HPV negative tumors compared to HPV positive tumors. CD4^+^/CD25^+^/CD127^low^ cells accounted for 11.5 ± 7.0% of T cells in HPV negative tumors and 8.1 ± 5.3% in HPV positive tumors (*p* = 0.24; Figure 4D, left plot). Similar results were obtained for the CD4^+^/CD39^+^ phenotype (28.8 ± 20.9% vs. 23.8 ± 7.8%; *p* = 0.52; Figure 4D, right plot).

Analyzing immune checkpoint expression of tumor-infiltrating T cells depending on HPV status revealed no significant differences between HPV negative and HPV positive tumors (Figure 4E, left plot). However, a trend towards higher percentages of PD-1^+^ T cells in the microenvironment of HPV positive HNSCC was observed compared to HPV negative tumors (48.4 ± 18.1% vs. 61.2 ± 14.1%; *p* = 0.12), due to significantly higher percentages of PD-1 expressing cytotoxic CD8^+^ T cells (75.4 ± 9.4% vs. 51.2 ± 24.6%, *p <* 0.05; Figure 4E, middle plot). No difference in PD-1 expression in CD4^+^ cells between HPV positive and HPV negative HNSCC (55.0 ± 16.4% vs. 49.1 ± 19.0%) was observed (Figure 4E, middle plot). A statistically not significant trend towards decreased proportions of CTLA-4 expressing T cells was observed in HPV positive HNSCC (6.6 ± 5.2% vs. 11.6 ± 8.5%; *p* = 0.17), which was similarly present in CD4^+^ and CD8^+^ T cells of HPV positive and HPV negative tumors (14.6 ± 11.6% vs. 9.9 ± 8.5%; *p* = 0.36; 8.3 ± 8.6% vs. 5.5 ± 4.2%; *p* = 0.44; Figure 4E, right plot).

### Regulatory T cells (CD4^+^/CD25^+^/CD127^low^) and the percentage of CTLA-4^+^ T cells are increased in the tumor microenvironment of early stage HNSCC

**Figure 5:**
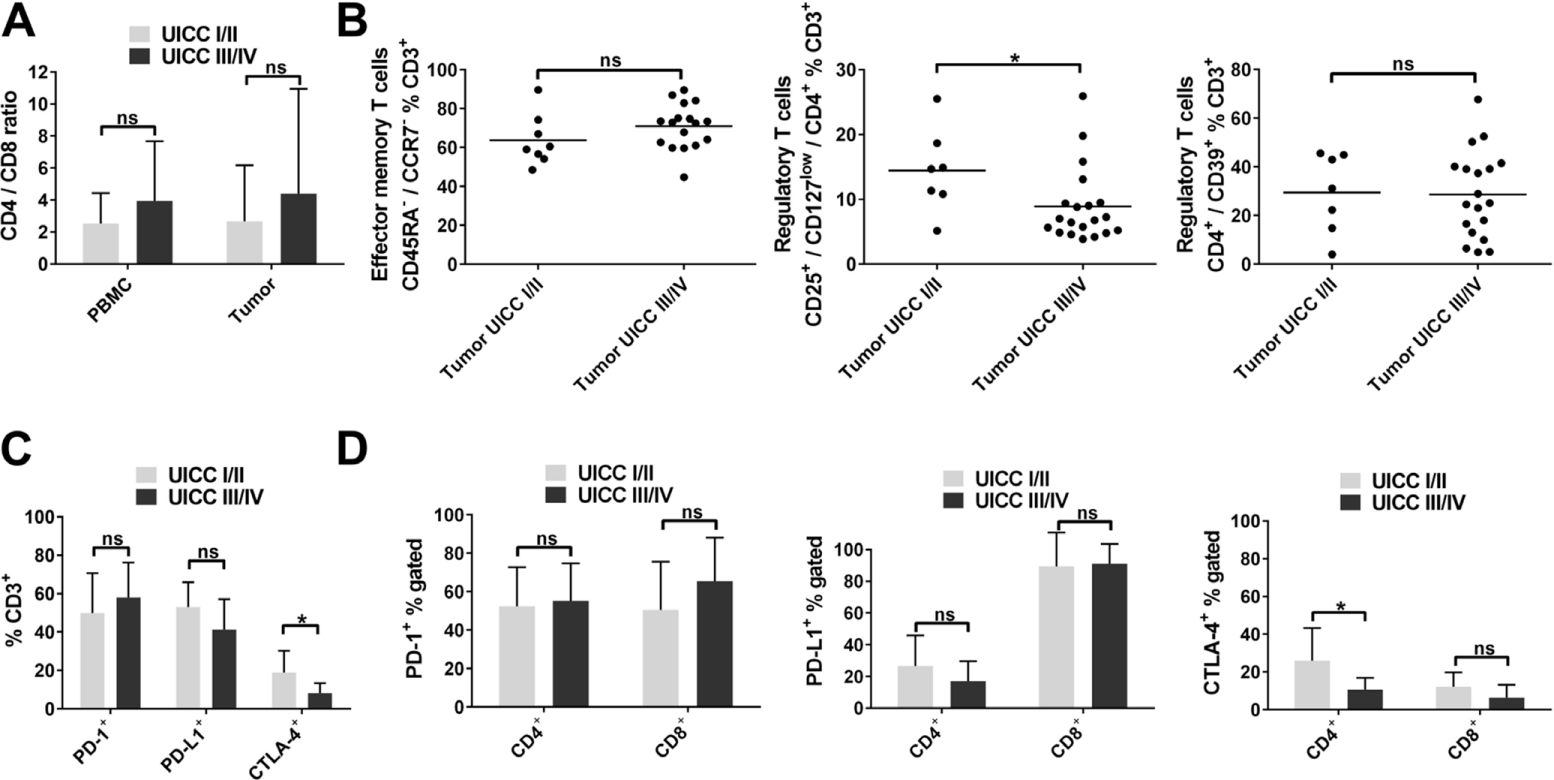
T-cell subsets in early and late stage HNSCC (**A**) CD4/CD8 ratio in PBMC and tumor tissue of HNSCC patients according to UICC tumor stage (stage I/II vs. III/IV). (**B**) Scatter plots showing effector memory T cells (CD45RA^−^/CCR7^−^; left) and regulatory T-cell phenotypes (CD4^+^/CD25^+^/CD127^low^; middle; CD4^+^/CD39^+^; right) in the tumor microenvironment of early (UICC I/II) and late (UICC III/IV) stage HNSCC. (**C**) Overview of checkpoint molecule expression (PD-1, PD-L1, CTLA-4) in tumor-infiltrating T cells in early and late stage HNSCC. (**D**) Comparison of percentages of PD-1^+^ (left), PD-L1^+^ (middle) and CTLA-4^+^ (right) cells in CD4^+^ and CD8^+^ T-cell subsets of early and late stage HNSCC tumors. PBMC and tumor UICC I/II, *n* = 11; PBMC and tumor UICC III/IV, *n* = 23; for statistical analysis, *t* tests were performed in (A) - (D), except for comparison of CTLA-4^+^ T cells in (C) and CTLA-4^+^% CD4^+^ in (D). Mann-Whitney test was used in these cases. Data is depicted as mean ± standard deviation. *P < 0.05; ns, not significant.

Patients with late stage HNSCC (UICC stage III/IV) demonstrated a slightly increased CD4/CD8 ratio compared to early stage tumors in PBMC and tumor microenvironment (3.9 ± 3.7 vs. 2.5 ±1.9; 4.4 ± 6.6 vs. 2.7 ± 3.5, respectively). However, mean values were not significantly different (*p* = 0.23; *p* = 0.51, respectively; Figure 5A). The majority of T cells within the tumor showed an effector memory phenotype irrespective of stage category (63.8 ± 13.1% vs. 70.9 ± 11.5%; Figure 5B, left plot). T cells with the regulatory phenotype CD4^+^/CD25^+^/CD127^low^ showed a higher percentage in the microenvironment of early stage HNSCC (14.4 ± 6.5%) compared to late stage HNSCC (8.9 ± 5.7%; *p <* 0.05; Figure 5B, middle plot). Percentages of T cells with the regulatory phenotype CD4^+^/CD39^+^ were similarly high (29.4 ± 16.3% vs. 28.6 ± 17.8%; Figure 5B, right plot).

No significant differences were observed regarding PD-1 and PD-L1 expression of tumor-infiltrating T cells related to disease stage (49.9 ± 20.8% vs. 57.9 ± 18.3%; *p* = 0.35 and 53.1 ± 12.9% vs. 41.2 ± 15.9%; *p* = 0.11). However, CTLA-4^+^ T cells were significantly decreased in the microenvironment of late stage HNSCC compared to early stage (18.9 ± 11.3% vs. 8.2 ± 5.2%; *p <* 0.05; Figure 5C), which was attributable to lower percentages of CTLA-4 expressing CD4^+^ T cells (10.6 ± 6.2% vs. 26.1 ± 17.2%; *p <* 0.05), but not to altered expression of CTLA-4 in CD8^+^ T cells (6.5 ± 6.7% vs. 12.2 ± 7.5%; *p* = 0.14; Figure 5D, right plot). PD-1 expression in the CD4^+^ and CD8^+^ T-cell subsets in HNSCC tumors was not significantly different comparing early and late stage (52.3 ± 20.3% vs. 55.1 ± 19.6% and 50.6 ± 25.0% vs. 65.3 ± 22.7%; Figure 5D, left plot). This was also the case for PD-L1 expression of tumor-infiltrating CD4^+^ and CD8^+^ T cells (26.5 ± 19.4% vs. 17.0 ± 12.6% and 89.4 ± 21.4% vs. 91.1 ± 12.5%, Figure 5D, middle plot).

## DISCUSSION

In this study, we provide the first comprehensive analysis of multiple T-cell subsets and the expression of different and clinically relevant immune checkpoint molecules on T cells in treatment-naïve HNSCC, non-cancerous mucosa and peripheral blood of patients and healthy donors. Previous studies investigating TILs and immune checkpoint molecules in HNSCC were mainly based on immunohistochemistry data [30–32]. Furthermore, our study combines the results of immunohistochemistry and flow cytometry. We found a significantly higher number of effector memory T cells and Treg in the tumor microenvironment compared to PBMC. Moreover, we could demonstrate that expression of the immune checkpoint molecules CTLA-4, PD-1 and PD-L1 was increased on intratumoral and circulating T cells in HNSCC patients compared to healthy donors, reflecting an immunosuppressive tumor microenvironment.

The lymphocytic infiltrate of tumors is known to have an impact on the prognosis of malignant disease [33–35]. In HNSCC, a high density of TILs is associated with improved patient outcome [36, 37]. PBMC of HNSCC patients were shown to consist of a significantly lower number of naïve CD4^+^/CD45RO^−^/CD27^+^ T cells and an increased pool of memory CD4^+^/CD45RO^+^ T cells compared to healthy controls [38]. In line with these results, we found a decreased rate of naïve T cells (CD45RA^+^/CCR7^+^) and an increased frequency of effector memory T cells (CD45RA^−^/CCR7^−^) in the tumor microenvironment compared to PBMC of cancer patients. However, the memory phenotype of T cells was also detected in non-cancerous mucosa. These findings could reflect changes in T-cell phenotypes while moving from peripheral blood into the tissue and the immunosuppressive microenvironment even in morphologically non-cancerous tissue.

Treg are increased in peripheral blood and tumor sites of patients with solid tumors including HNSCC [16, 39, 40], which contributes to the suppression of an effective antitumor immune response. Our results support previous findings by showing an increased rate of Treg (either CD4^+^/CD25^+^/CD127^low^ or CD4^+^/CD39^+^) in TILs compared to non-cancerous mucosa and PBMC of tumor patients. Furthermore, the fraction of CD4^+^/CD25^+^/CD127^low^ Treg was increased in PBMC of patients compared to healthy controls, suggesting a systemic immunosuppressive activity in HNSCC patients. Surprisingly, the same Treg phenotype was elevated in the microenvironment of low stage HNSCC compared to advanced stage. This potentially reflects a reduction of tumor-induced inflammation in early disease mediated by Treg [41].

Immune checkpoint molecules including CTLA-4, PD-1 and PD-L1 are key molecules involved in maturation and suppressive functions of tumor-infiltrating T cells [23, 24]. Immune checkpoint molecules are characteristic of exhausted T cells [42], which develop after long-term exposure to antigens [43]. In consequence, exhausted T cells are incapable of robust effector responses upon antigen exposure [44, 45]. However, they can be reactivated using immune-checkpoint blockade [46]. In HNSCC, PD-1^+^ T cells were found to be increased and associated with an improved prognosis in the setting of HPV positivity [40, 47, 48]. In a recent study evaluating PD-L1 expression by immunohistochemistry in oral squamous cell carcinoma, high PD-L1 levels were associated with metastasis and poor prognosis [49]. PD-1-pathway blockade in tumors lacking PD-L1 expression on tumor cells resulted in clinical response in different entities including HNSCC [25, 50, 51]. This phenomenon can be explained by expression of PD-1 or PD-L1 on non-malignant cells in the tumor microenvironment. Expression of immune checkpoints on primary tumor cells has been analyzed in different cancer entities. However, their expression on TILs is widely unknown. Our study represents the first comprehensive analysis of immune checkpoint expression on tumor-infiltrating T-cell subsets in HNSCC, non-cancerous mucosa and peripheral blood from patients and healthy donors. We detected a significantly higher rate of PD-1 and CTLA-4 expression on TILs compared to PBMC of cancer patients with highly variable expression profiles on CD4^+^ and CD8^+^ cells. Strikingly, the percentage of PD-1, PD-L1 and CTLA-4 expressing circulating T cells was increased in HNSCC patients compared to healthy donors, again pointing towards elevated proportions of regulatory or exhausted T-cell phenotypes in HNSCC patients. Taken together, these results clearly demonstrate the importance of a comprehensive analysis of immune checkpoint expression on tumor-infiltrating and circulating lymphocytes to identify their impact on response to treatment in future studies targeting these pathways.

Recently, differences in the immune infiltrate between HPV positive and HPV negative tumors have been described. HPV positive tumors are characterized by an adaptive host immune response directed against viral antigens as well as specific T cells directed against viral proteins [4, 52, 53]. Further studies demonstrated a higher number of CD8^+^ T cells and a decreased CD4/CD8 ratio in p16-positive patients [48, 53]. We found a significantly higher number of CD45^+^ lymphocytes in HPV positive HNSCC than in HPV negative tumors. This finding may reflect the adaptive immune response against HPV antigens as well as a tumor-specific immune response. However, the relative proportion of T-cell subsets did not significantly differ between HPV positive and HPV negative tumors. There was a trend towards a higher number of tumor-infiltrating Treg in HPV negative tumors, which might correspond to a more immunosuppressive tumor microenvironment in HPV negative tumors. In this study, certain differences did not reach significance or might have been missed due to the number of analyzed PBMC or tumor samples (*n* = 34) and especially the highly heterogeneous distribution of T-cell subsets and immune checkpoint molecule expression.

PD-1 expression in TILs of HPV positive tumors was higher than in HPV negative tumors. This finding might reflect the fact that viral proteins induce a strong immune response represented by tumor-infiltrating memory T cells and PD-1^+^ T cells. This hypothesis is supported by recent data showing higher expression of PD-1 mRNA in HPV positive tumors [48]. However, a recent study also demonstrated high levels of PD-1 and PD-L1 expression in HPV negative HNSCC [54]. Our results confirm previous findings that PD-L1 expression in TILs does not differ between HPV positive and HPV negative patients [55]. CTLA-4 expression in HPV positive HNSCC was lower than in HPV negative tumors. This is in line with the finding that regulatory T cells, as highly CTLA-4 expressing cells were similarly less frequent in HPV positive tumors.

Clinical trials applying anti-PD-1 inhibitors showed activity regardless of HPV status in HNSCC [25, 56]. Since we found elevated checkpoint molecule expression in HPV positive and negative HNSCC, our results strongly support further evaluation of immune checkpoint inhibitors regardless of HPV status. Analysis of tumor-associated lymphocytes in HNSCC patients treated with immune checkpoint inhibitors could give further insights into the mode of action and factors determining response to these treatments. Recent studies in melanoma patients also suggest that the ‘Immunoscore’ can be a useful tool in predicting response to checkpoint inhibition [57]. In view of this finding, we established an automated analysis covering the entire cross section of HNSCC tumors and investigating CD3^+^ and CD8^+^ cell densities in HNSCC. Given the often highly heterogeneous immune cell infiltration in different areas of the same HNSCC, the entire area of tumor core and invasive margin in one slide was included. In our opinion, this provides more reliable and consistent results than the analysis of tissue microarrays, which use only small, potentially not representative proportions of the tumor. Results were highly concordant with flow-cytometric analysis of the same tumors. In HPV negative HNSCC, high CD3^+^ and CD8^+^ cell infiltration was mainly observed in tumors with increased levels of MHC class I expression on tumor cells, suggesting a recognition of MHC I-presented tumor antigens by infiltrating T cells, which is supported by previous findings that TILs from HNSCC comprise tumor-specific T cells [58]. This is particularly important, since response to checkpoint inhibitors is related to pre-existing antitumor T-cell activity [59]. Whether the ‘Immunoscore’ and especially the combination with flow-cytometric analyses, has prognostic relevance and a predictive value regarding immunotherapy of HNSCC will be in the focus of future investigations.

Taken together, our results show a substantial rate of regulatory T cells and expression of immune inhibitory checkpoint molecules reflecting an immunosuppressive environment in HNSCC. HPV status is not related to alterations of T-cell subsets or profound differences in immune checkpoint molecule expression. In-depth analysis of tumor-associated immune cells and checkpoint molecule expression could prove useful in clinical routine in the future, especially since reliable predictive markers for treatment response to immune checkpoint inhibitors are still lacking.

## MATERIALS AND METHODS

### Patient characteristics

Tumor samples (*n* = 34), peripheral blood mononuclear cells (PBMC; *n* = 34) and random biopsies from macroscopically non-cancerous mucosa (*n* = 7) of 34 patients with diagnosed squamous cell carcinoma of the head and neck were obtained between 2013 and 2016. Importantly, all samples were acquired before any kind of anticancer therapy was initialized. Tumor stage was assessed according to the UICC tumor-node-metastasis criteria (7th edition). Peripheral blood samples of 15 healthy donors were included as controls for PBMCs. The patient characteristics are summarized in Table 1. Written informed consent was obtained from all patients prior to surgery and from all healthy donors. Our institutional review board approved the study (protocol no. 11-116).

### Cell isolation from human peripheral blood and tumor

Peripheral blood was obtained from patients and age- and sex-matched healthy donors immediately prior to surgery. PBMC were purified using density-based separation with Pancoll Human (PAN-Biotech, Cat. No. P04-60100). Fresh unfixed tissue from primary tumors or non-cancerous mucosa, which was not required for pathological analyses, was processed immediately after tumor biopsy or surgical resection. Fresh tissue was manually minced and transferred into single cells suspensions using a gentleMACS Dissociator (Miltenyi) with DNase I (100U/mL, Applichem; Cat. No. A3778) and Collagenase IV (320u/mL, Worthington; Cat. No. LS004180). Cells were filtered through 100 μm and 70 μm nylon cell strainers (Greiner Bio-One; Cat. No. 89508-344 and 89508-340).

### Flow cytometry

At least 5 × 10^5^ events per sample were acquired on a Gallios 10-color flow cytometer (Beckman Coulter). Phenotypic characterization of T-cell subsets was performed using CD3-APC-Cy7, CD39-APC, CTLA-4-PE (all BD Biosciences), CD4-PerCP/Cy5.5, CD8-PE/Cy7, PD-1-APC, PD-L1-PE/Cy7, CCR7-FITC, CD25-AF700, CD127-PerCP/Cy5.5, CD45RA-AF700 (all Biolegend), CD45-PE-eFluor610 (eBioscience) and aqua dead cell stain (Life Technologies).

### Immunohistochemistry

Immunohistochemical staining was performed on formaldehyde-fixed, paraffin-embedded tissue sections of 3 μm thickness using monoclonal mouse anti-human primary antibodies for MHC class I (Clone EMR8-5; Abcam; Cat. No. AB70328), CD8 (Clone C8/144B; Dako; Cat. No. M710301-2) and P16^INK4A^ (Clone G175-405; BD Biosciences; Cat. No. 550834) and rabbit anti-human antibody for CD3 (Thermo Scientific; Cat. No. MA1-90582) followed by appropriate biotinylated secondary antibodies. Every analysis contained negative and positive controls. Detection of secondary antibodies was achieved by avidin-biotin-peroxidase complex (ABC) method using the Vectastain-Elite-ABC kit (Vector Laboratories; Cat. No. PK-6100). Diaminobenzidine tetrahydrochloride/H_2_O_2_ was used to visualize peroxidase activity, hematoxylin as nuclear counterstaining. Strong cytoplasmic and nuclear staining was regarded positive for p16 expression. MHC class I expression was classified as high, if more than 80% of tumor cells showed strong staining, otherwise it was classified as low MHC class I expression.

### DNA isolation, HPV-DNA detection by PCR and HPV typing

DNA was extracted from paraffin-embedded tissue samples using the Gentra Puregene Tissue kit with proteinase K treatment (Qiagen) according to the manufacturer's protocol.

Nested PCR was performed to detect HPV DNA as described previously [60]. After purification (QIAquick PCR Purification kit, Qiagen; Cat. No. 28104), A6/A8 and GP5^+^/GP6^+^ PCR products were sequenced (LIGHTRUN™) by GATC Biotech using the A6 or GP5^+^ primer, respectively. HPV subtypes were identified by comparing the sequencing results to the NCBI database entries (NCBI BLAST alignment).

### Automated analysis of CD3/CD8 cell density

FFPE (formalin-fixed, paraffin-embedded) sections covering the whole cross section of the primary tumor including sufficient adjacent tissue from 16 surgically treated HNSCC patients were selected for immunohistochemical analysis of CD3 and CD8. High-resolution images (20x objective) were captured using a SCN400 slide scanner (Leica Biosystems). Image pre-processing was performed with Aperio ImageScope software (Leica Biosystems) and GNU Image Manipulation Program (version 2). Tumor borders were delineated by an experienced pathologist. The area of the invasive margin (IM) was determined as ranging from 50 μm within the tumor measured from the tumor border to 300 μm outside the tumor border. Tumor core (CT) comprised the whole tumor section excluding the first 50 μm adjacent to the tumor border. Necrotic areas were excluded from analysis. IM and CT sections were separated and cropped into tiles of 1024×1024 pixels utilizing OpenSlide Python and the libvips image processing library [61, 62]. Automated analysis of DAB-positive cells per area was performed using CellProfiler software (version 2.1.1). A schematic overview of the analyzing process is depicted in Figure 2B. Counting accuracy was verified by optical counting of selected tissue areas. Using the median CD3^+^ or CD8^+^ cell density of all analyzed samples as cut-off value, samples were classified as CD3^high^ or CD3^low^ and CD8^high^ or CD8^low^, respectively. IM and CT were analyzed separately. Subsequently, tumors were subdivided into five groups (0-IV) according to their CD3^+^ and CD8^+^ cell infiltration of IM and CT as described previously [20]. Subgroups 0-I were termed ‘Immunoscore low’, III-IV ‘Immunoscore high’.

### Data analysis

Unless otherwise stated, data is shown as mean ± standard deviation. Samples with cell count below a threshold of 500 detected T cells per analysis were excluded from further subset analysis. Flow cytometry data was analyzed using the Kaluza Software (Version 1.1, Beckman Coulter) and GraphPad Prism 7 (GraphPad Software). Shapiro-Wilk test and Levene's test were applied to test for normality and homoscedasticity, respectively. In case of normal distribution and homoscedasticity, one-way ANOVA was performed to compare independent measures of more than two groups and Student's *t*-test for comparison of two groups. Otherwise, non-parametric Mann-Whitney or Kruskal-Wallis tests were used for comparisons of two or more than two groups, respectively. Fisher's exact test was applied to test for association between categorical variables.

## SUPPLEMENTARY MATERIALS FIGURE



## Abbreviations

ABC: avidin-biotin-peroxidase complex
ANOVA: analysis of variance
CT: tumor core
CTLA-4: cytotoxic T-lymphocyte-associated antigen 4
DAB: 3,3′-diaminobenzidine
DNA: deoxyribonucleic acid
FFPE: formalin-fixed, paraffin-embedded
HC: healthy control
HNSCC: head and neck squamous cell carcinoma
HPV: human papillomavirus
IM: invasive margin
MHC: major histocompatibility complex
mRNA: messenger ribonucleic acid
PBMC: peripheral blood mononuclear cells
PCR: polymerase chain reaction
PD-1: programmed cell death 1
PD-L1: programmed cell death 1 ligand 1
TIL: tumor-infiltrating lymphocytes
Treg: regulatory T cells
UICC: Union for International Cancer Control
