# Supplementary material for: Characterization of tumor-associated T-lymphocyte subsets and immune checkpoint molecules in head and neck squamous cell carcinoma
Source: Oncotarget. 2017 May 16;8(27):44418–33. doi: 10.18632/oncotarget.17901 (PMC5546490; doi:10.18632/oncotarget.17901)
Supplement: Supplementary file 1 [file oncotarget-08-44418-s001.pdf]

Characterization of tumor-associated T-lymphocyte subsets and immune checkpoint molecules in head and neck squamous cell carcinoma

Supplementary Materials

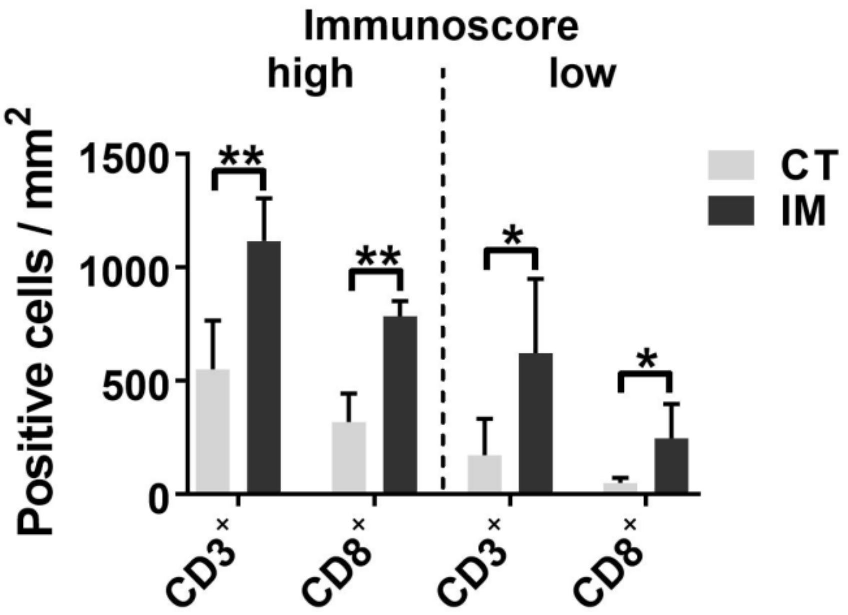

Supplementary Figure 1: CD3$^+$ and CD8$^+$ cell densities in 'Immunoscore high/low' HNSCC. Results of automated analysis of CD3 and CD8 immunohistochemistry are summarized. CD3$^+$ and CD8$^+$ cell numbers per mm^2 in tumor core (CT) and invasive margin (IM) of tumors classified as 'Immunoscore high' and 'Immunoscore low' are shown in bar graphs (mean value and standard deviation). For statistical analysis, t tests were performed. Data is depicted as mean ± standard deviation. **P* < 0.05; ***P* < 0.005.
